# Trauma and Violence Informed Care Through Decolonising Interagency Partnerships: A Complexity Case Study of Waminda’s Model of Systemic Decolonisation

**DOI:** 10.3390/ijerph17207363

**Published:** 2020-10-09

**Authors:** Patricia Cullen, Tamara Mackean, Faye Worner, Cleone Wellington, Hayley Longbottom, Julieann Coombes, Keziah Bennett-Brook, Kathleen Clapham, Rebecca Ivers, Maree Hackett, Marlene Longbottom

**Affiliations:** 1 School of Population Health, University of New South Wales Sydney, Samuels Building, Kensington, NSW 2033, Australia; rebecca.ivers@unsw.edu.au; 2 The George Institute for Global Health, University of New South Wales Sydney, Newtown, NSW 2042, Australia; tmackean@georgeinstitute.org.au (T.M.); jcoombes@georgeinstitute.org.au (J.C.); kbennett-brook@georgeinstitute.org.au (K.B.-B.); mhackett@georgeinstitute.org.au (M.H.); 3 Ngarruwan Ngadju: First Peoples Health and Wellbeing Research Centre, University of Wollongong, Wollongong, NSW 2522, Australia; kclapham@uow.edu.au (K.C.); marlenel@uow.edu.au (M.L.); 4 College of Medicine and Public Health, Flinders University, Bedford Park, SA 5042, Australia; 5 Waminda South Coast Women’s Health and Welfare Aboriginal Corporation, Nowra, NSW 2541, Australia; Faye@waminda.org.au (F.W.); CleoneWellington@waminda.org.au (C.W.); HayleyLongbottom@waminda.org.au (H.L.)

**Keywords:** First Nations, Indigenous, Aboriginal, decolonisation, racism, primary health, culturally safe, trauma and violence informed care, complexity theory, whiteness

## Abstract

Through the lens of complexity, we present a nested case study describing a decolonisation approach developed and implemented by Waminda South Coast Women’s Health and Welfare Aboriginal Corporation. Using Indigenous research methods, this case study has unfolded across three phases: (1) Yarning interviews with the workforce from four partner health services (*n* = 24); (2) Yarning circle bringing together key informants from yarning interviews to verify and refine emerging themes (*n* = 14); (3) Semi-structured interviews with a facilitator of Waminda’s Decolonisation Workshop (*n* = 1) and participants (*n* = 10). Synthesis of data has been undertaken in stages through collaborative framework and thematic analysis. Three overarching themes and eight sub-themes emerged that centred on enhancing the capabilities of the workforce and strengthening interagency partnerships through a more meaningful connection and shared decolonisation agenda that centres Aboriginal and Torres Strait Islander families and communities. Health and social services are complex systems that function within the context of colonisation. Waminda’s innovative, model of interagency collaboration enhanced workforce capability through shared language and collective learning around colonisation, racism and Whiteness. This process generated individual, organisational and systemic decolonisation to disable power structures through trauma and violence informed approach to practice.

## 1. Introduction

Indigenous peoples globally are spiritually and culturally diverse. Yet for those whose land has been colonised, there is a shared universality in experiences of survival, dispossession, systemic racism and colonial oppression [1]. Aboriginal and Torres Strait Islander people are the first peoples of Australia and the world’s oldest surviving continuous culture for more than 60,000 years. As a colonised nation, Australia’s history marks immeasurable pain and trauma for Aboriginal and Torres Strait Islander peoples, and the ongoing legacy of colonisation has disrupted Aboriginal and Torres Strait Islander ways of knowing, being and doing [2,3,4,5,6,7]. At its core, colonisation is a violent act. Contemporary manifestations of colonial violence persist in overt forms (e.g., interpersonal racism) and through structural violence (e.g., systemic and institutional racism), which Aboriginal and Torres Strait Islander peoples experience disproportionately in Australia [7].

For many Aboriginal and Torres Strait Islander peoples, these historical and present experiences of violence manifest as situational, cumulative and transgenerational trauma with profound impacts on health and wellbeing. Transgenerational trauma results from exposure to trauma that may be transferred from the first generation that have experienced (or witnessed) violent or traumatic events to the second and further generations of descendants [8]. In Australia, the dominant discourse centres on Aboriginal and Torres Strait Islander peoples’ health in terms of disparity, disadvantage and deficit, which does not give adequate attention to structural determinants, transgenerational trauma and present trauma, including experiences of racism [9]. Racism has been linked to poor health outcomes and is a barrier to accessing health care among Aboriginal and Torres Strait Islander people in Australia [3,10,11,12,13,14]. While policies and interventions generally acknowledge the ongoing legacy of colonisation, this does little to actively redress the contemporary manifestations of colonisation that persist in the form of transgenerational trauma, structural inequity and intersecting forms of racism in Australia. 

Racism sits within the context of Australia as a colonised country, in which Whiteness is “the invisible norm against which other races are judged in the construction of identity, representation, decision-making, subjectivity, nationalism, knowledge production and the law” [15] (p. 388). Aileen Moreton-Robinson’s work has been critical in advancing the theory in Australia around Whiteness and the relationship with Indigenous sovereignty. Drawing on critical Whiteness literature, Moreton-Robinson describes white possessive logic as the “possessiveness” of white supremacy, which is endemic in Australian society and is evident in language describing First Nations people as “our Indigenous people” or “Australia’s Indigenous people”. Lack of awareness and resistance to acknowledging the dominance of white possessive logic feeds into societal acceptance of ideology and historical inaccuracies around colonisation that maintain hegemonic Whiteness in Australia. 

Recognition of the role hegemonic Whiteness plays in constructing and perpetuating white possessiveness sits alongside a shift toward decolonisation of policy and practice. Decolonisation requires “all individuals to explore their own assumptions and beliefs so that they can be open to other ways of knowing, being, and doing” [6] (p. 188). Dudgeon and Walker [5] (p. 291) explain that we experience the world through a colonised lens and an essential part of decolonisation is “recognising how we have all been affected by colonisation”. In practice, decolonisation entails actions toward redressing colonising practices and recognising that colonisation has and continues to impact Indigenous and non-Indigenous people [3,5,16].

In the health sector, Sherwood and Edwards [6] (p. 180) assert that decolonisation requires all health workers to understand and acknowledge the context of colonisation as a necessary step in understanding the context of Aboriginal and Torres Strait Islander peoples’ health. This recognition of context also requires rethinking the drivers of health and wellbeing, acknowledging the holistic nature of health, and working collaboratively—led by First Nations people—toward remediation rather than problematising Aboriginal and Torres Strait Islander peoples and their health. To this end, Aboriginal Community Controlled Health Organisations (ACCHOs) seek to decolonise health care delivery for the clients within their service, but also more broadly in their communities to counter systemic and institutional racism and minimise the traumatic impacts for their clients and communities. 

It is within this context that the First Response project arose. First Response is a research partnership with four ACCHOs in New South Wales (NSW), the University of New South Wales and University of Wollongong and is funded by the Lowitja Institute. The four partner ACCHOs include (1) Waminda South Coast Women’s Health and Welfare Aboriginal Corporation, located on the South Coast of NSW on Yuin Country; (2) Katungul Aboriginal Corporation Regional Health and Community Services, located on the far South Coast of NSW on Yuin Country; (3) Illawarra Aboriginal Medical Service on Dharawal Country; and (4) Yerin Aboriginal Health Services, located on the NSW Central Coast on Darkinjung Country. The First Response research team is comprised of eight senior, mid and early career researchers. Five researchers are Aboriginal or Torres Strait Islander women, of whom four are connected to the communities of the First Response partner ACCHOs. First Response includes qualitative interviews with the workforce as well as a scoping review of policy responses to understand how primary health services respond to ongoing impacts of trauma, violence and institutional racism through integration of trauma and violence informed care [17].

Trauma and violence informed care centres on understanding the context in which peoples’ health is experienced and recognising how this intersects with interpersonal violence, structural violence, inequity and trauma, including trauma attributed to colonisation and racism [18]. It is distinct from Western notions of trauma that centre on individual’s experiences of traumatic events, which has been critiqued as a reductionist lens that does not adequately encompass First Nations peoples’ experiences of trauma and violence [17]. A key tenet of trauma and violence informed care is that trauma is considered holistically, encompassing the transgenerational effects of trauma as well as situational, cumulative and historical trauma [17,18]. Historical trauma is the “subjective experiencing and remembering of events in the mind of an individual or the life of a community, passed from adults to children in cyclical processes” [8] (p. 138). In addition, trauma and violence informed care seeks to address and acknowledge structural and interpersonal violence as key determinants of health [17]. In this approach, violence encompasses experiences of interpersonal violence (of physical, emotional, sexual, social, spiritual, cultural, psychological and economic abuses that occur within families, intimate relationships, extended families, kinship networks and communities) [19], as well as structural violence inflicted by policies and institutional practices that create and perpetuate harm through unequal and unjust social conditions. In this way, the core components of trauma and violence informed care are aligned with decolonisation, it is regardful of people’s experiences and aims to minimise or prevent retraumatisation [18].

An important finding from *First Response* is that trauma and violence informed care is vital and the ACCHO workforce play a key role in supporting people and families both within ACCHOs and in the wider community. One of the ways that Waminda had addressed this need was through workforce and interagency decolonising approaches that center Aboriginal and Torres Strait Islander families and communities. Within the existing research partnership Waminda approached the research team to partner in this case study inquiry into how ACCHOs integrate trauma and violence informed care through systemic decolonisation. We explore this using a nested case study describing a workforce and interagency partnership approach developed and implemented by Waminda South Coast Women’s Health and Welfare Aboriginal Corporation in NSW, Australia.

## 2. Materials and Methods

### 2.1. Design and Theory

**Figure 1:**
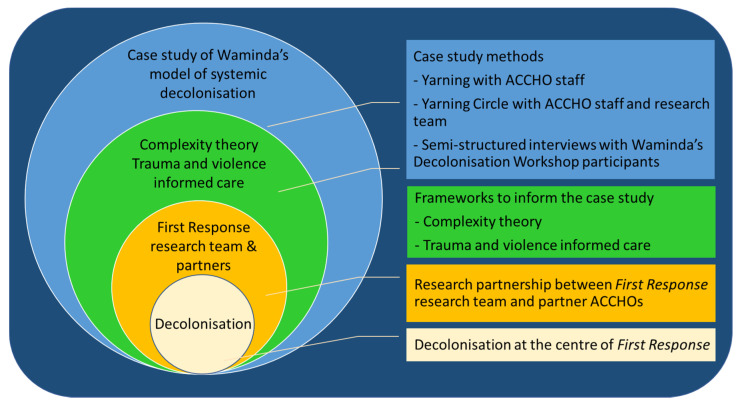
The case study is informed synergistically by complexity theory and trauma and violence informed care, which has been undertaken within the existing research partnership of the First Response project, in which decolonisation is central. The case study brings together yarning with staff from the partner Aboriginal Community Controlled Health Organisations (ACCHOs) and semi-structured interviews with the Decolonisation Workshop participants.

Decolonisation is at the centre of the First Response project and the nested case study (Figure 1) [20]. As such, our theoretical stance draws on Indigenous scholarship around decolonisation, which assert that dominant ways of knowing, being and doing are grounded in white superiority and are the products of historical inaccuracies and institutionalised practices and identities [7].

Our approach to this case study is informed by complexity theory, which depicts health services as functioning within an interconnected ecological system that cannot be considered separately from its environment [20,21]. In health systems research, complexity theory depicts the interdependencies and interactions between system elements (e.g., individuals, collectives or processes) and the processes of “self-organisation” into a dynamic integrated system [22,23,24,25]. This approach has recently been used in the context of primary health care responses to violence against women in New Zealand [25] and in primary health settings in Canada that are trauma and violence informed [26]. Additionally, an over-arching framework throughout the First Response project has been informed by the principles of trauma and violence informed care [18]. Synergistically, these concepts of have shaped our approach to collecting, analysing and synthesising the data.

### 2.2. Ethics, Participants and Data Collection

Ethics approval was received from the NSW Aboriginal Health and Medical Research Committee 5th February 2018 (Project Reference number 1368/18). 

Phase 1: Yarning interviews with the primary health care workforce at four partner ACCHOs (*n* = 24; May–July 2018). Participants included eleven staff who identified as Management or Team leaders and thirteen clinical and allied health team members including Aboriginal and Torres Strait Islander Health Workers and Practitioners, General Practitioners, Nurses, Midwives, Mental Health Nurses as well as the Integrated Care, Social and Emotional Wellbeing, and Drug and Alcohol teams. Yarning is an Indigenous research method that takes a conversational approach to data collection, promotes cultural safety and positions the interviewee as the expert in constructing their own story rather than being a didactic researcher-driven process [27,28,29]. Importantly, yarning is deeply rooted in storytelling and oral traditions that are important ways of sharing knowledge for Aboriginal and Torres Strait Islander peoples, and through yarning the researcher builds a “relationship that is accountable to Indigenous people participating in the research” [27] (p. 38). Yarning is conversational, and rather than following a question and answer format of semi-structured interviews, can shift between different types of yarning, including the social yarn, the therapeutic yarn and the research yarn [27]. Yarning in research can move between these different types of yarns [27]. The social yarn is very informal, often meandering and occurs before the research yarn as an essential component to building trust between the researcher and the participant [27]. The research yarn is where the stories shared are focused on the research topic, and while informal it also has purpose (to obtain information) and structure (a defined beginning and end) [27]. The therapeutic yarn is not counselling, but is initiated when a participant shares information that is emotional or distressing and the researcher shifts toward actively supporting the participant, affirming their story and working with them to make meaning or understand their experience in new ways [27]. 

The research team have all undertaken training in yarning methods with Professor Dawn Bessarab, who is is a Bard/Yjindjabandi woman and an expert in Indigenous research, qualitative methodologies and yarning methods [27]. Yarning interviews were undertaken by three members of the research team (M.L., J.C., P.C.) to explore staff experiences and insight into trauma and violence informed care, partnerships with other services and sectors, as well as any areas that they would like to develop and resources that would support their work.

Phase 2: Yarning circle bringing together key informants from Phase 1 and the research team to verify emerging themes and further explore issues that were put forward by participants (*n* = 14). The one-day yarning circle was held in October 2018 on Gadigal Country in Sydney NSW; this included a collaborative yarning circle and a digital scribe to develop graphical representation of the main themes and issues. Collaborative yarning is another type of yarning that occurs between two or more people, in which ideas and information are shared in a way that can lead to new understandings and insights [27]. The collaborative yarning circle was a space for learning and sharing between staff from ACCHOs and the research team—in this space, we collectively delved into themes from the yarning interviews and also determined whether, and how, some of the issues identified resonated across ACCHOs.

Phase 3: Focused on the decolonisation work that Waminda has led in the Illawarra Shoalhaven region, including a Decolonisation Workshop facilitated by Waminda in October 2019. The workshop was attended by regional leaders from across the Southern region who were representing their organisations as part of the Illawarra Shoalhaven Department of Communities and Justice Strategic Leaders Forum. The workshop was facilitated by six staff from Waminda and two Dharawal Elders, and was delivered through formal presentations, sharing of personal and community stories and small group breakout sessions for attendees to consolidate understanding and engage in interactive learning. The content of the workshop draws widely on Australian and international scholarship, local knowledge as well as family and personal histories, covering: Colonisation, local history, racism, white privilege and fragility.

A member of the research team (P.C.) attended this workshop to observe the content of the workshop and how it was delivered. However, it was important for the integrity of the workshop that participants were comfortable to interact freely and without concerns that their dialogue or responses would be reported. Therefore, no recordings were made, and participants’ dialogue from the workshop is not reported.

Following the workshop, attendees were invited to participate in a semi-structured interview. The semi-structured interviews were undertaken with a facilitator of Decolonisation Workshop from Waminda (*n* = 1; July 2019) and participants (*n* = 10; November-December 2019) by phone by a member of the research team (P.C.). Interviews explored their perspectives on the following: (1) Trauma and violence informed care; and (2) The Decolonisation Workshop and how this may impact their work with Aboriginal and Torres Strait Islander families, the interview guide is included in the Supplementary Materials.

### 2.3. Analysis and Synthesis

All semi-structured interviews, yarning interviews and the yarning circle were audio recorded with permission and professionally transcribed. In addition, collaborative yarning among the research team was recorded as reflective research notes by P.C., T.M. and M.L. The reflective research notes along with the research data were managed using NVivo 12 software [30]. Iterative triangulation and thematic analysis of these data was collaboratively undertaken in line with a decolonisation approach [31]. Importantly, thematic analysis embraces researcher subjectivity and reflexivity, such that the generation of themes is an active process and themes do not passively emerge from the data [32]. 

Thematic analysis firstly involved reading a selection of the transcripts by an Aboriginal researcher (M.L.) and a non-Aboriginal researcher (P.C.) who then developed the initial coding framework in alignment with the principles of trauma and violence informed care and decolonisation, and used this to code the remaining transcripts deductively [33]. As an Aboriginal researcher (M.L.) and non-Aboriginal researcher (P.C.) who both come from the South Coast of NSW where this case study was situated, this process involved much reflection on how and where our experiences shaped our perspectives of the data. Specifically, our experiences and perspectives of whiteness, racism, white privilege, and multiple forms of violence and trauma were often at the forefront of these reflections. Through this reflexive praxis, our divergent histories and standpoints were critical in understanding and interpreting the data drawn from the perspectives of non-Aboriginal participants and Aboriginal and Torres Strait Islander participants, which shaped the coding framework. Through an iterative process, the codes were amalgamated into higher order concepts, and then these were shaped into themes.

The resonance of this initial analysis was shared with the research team and then refined through collaborative yarning. This process involved P.C. and M.L. sharing the codes and themes with relevant data so that the research team could reflect, question and shape emerging themes. The themes were further refined through collaborative yarning in the yarning circle, such that the emerging themes, with examples from the data, were shared and explored with staff from the partner ACCHOs. The collaborative yarning allowed for in-depth interpretations of the data, as experiences and reflections were shared between staff from the partner ACCHOs. Reflective and descriptive notes made by the research team at all stages of the collaborative yarning were drawn on to refine and shape the meaning of themes. 

The final stage of analysis took place following the semi-structured interviews with the decolonisation workshop participants. The coding framework was applied by P.C. and T.M., and new themes were developed through iterations of amalgamating codes into higher order concepts and then themes. These themes were shared and refined with research partners from Waminda through collaborative yarns. In the final stage of synthesis, the themes derived from the yarning interviews and circle were brought together with the themes derived from the semi-structured interviews to depict Waminda’s approach to systemic decolonisation. 

Overall, researcher reflection and standpoint, which are fundamental elements of both thematic analysis and collaborative yarning have been central to our analysis, interpretation, and synthesis of results. This process brings together Indigenous research methods with qualitative research and case study methods, such that the collaborative analysis embraces diverse world views through a decolonisation lens and embraces multiple standpoints.

## 3. Results

The data were separated into three overarching themes according to the evolution of Waminda’s decolonisation approach over time: (1) complexity of interagency partnerships; (2) a new approach to decolonising the workforce; (3) decolonising partnerships. Across these three themes, the data were amalgamated into eight sub-themes. 

### 3.1. Complexity of Interagency Partnerships 

ACCHO staff identified that effective partnerships and working relationships with other services, both government and NGOs, are essential to achieving good outcomes for their clients and communities. Describing the complexity of interagency partnerships was key to understanding the system context and how this sits within the dynamic of colonisation. Three key sub-themes emerged relating to the context of how interagency partnerships function: (1) why advocacy; (2) making partnerships work; (3) acts of resistance.

#### 3.1.1. Why Advocacy

Advocacy by ACCHO staff was evident at all levels—on behalf of individual clients or families, on behalf of their organisation and at a policy level around funding, programs and key performance indicators (KPIs). ACCHO staff clearly stated that partnerships and integration with other services is an essential part of delivering effective health care. There was a sense that clients and families frequently “miss out on” essential services due to the complexity of navigating multiple services across several sectors, particularly in the face of excessive bureaucracy and pragmatic barriers such as transport, cost and literacy.

*“And that’s not saying we should be duplicating services, or just doing the work of other services but it’s just making sure that people don’t miss out really”*

(ACCHO Management/Team leader)

Staff described their role as advocates for communities and clients, and a major component of advocacy was breaking down information for clients to facilitate awareness of options available, agency to make informed decisions and access appropriate supports.

*“[I] do a lot of advocating for people to government departments such as Centrelink and Department of Housing; and now with the NDIS (National Disability Insurance Scheme) I assist people with their applications”*

(ACCHO Team member)

*“There’s a lack of information for clients, a big lack of information for clients and I know even since I’ve been down here in 10 years the information that’s supplied by other services, and even government organisations, is very confusing for victims and their children and they don’t understand it so they don’t follow through with it because they don’t have the understanding of what’s going on, that’s the difference I think”*

(ACCHO Management/Team leader)

ACCHO staff viewed their role in supporting clients to navigate and engage with external services as important to mitigating negative interactions and experiences that clients frequently encountered in government and non-government organisations. Staff reported that other services were often not culturally safe for clients, and that those services’ staff lacked cultural competence and were at times overtly racist or dismissive of clients.

*“So, it is very different, we do work in that strength-based way, which is very different to pretty well everyone else to be honest. But that’s complex because, you know, the women get a particular experience here. I’m not saying we’re perfect by any means but if you get a particular way of working here and then you’re trying to navigate a whole heap of other systems or people or workers who say, well you know, you were late or you don’t give a shit because you didn’t turn up again. And, you know, how many chances do you need”*

(ACCHO Management/Team leader)

*“I’m really supportive of the police and the hospital system, I understand their jobs are really hard but that’s probably the biggest challenge. So, when crunch comes to crunch and you have quite an acute issue I find the response—especially when it’s repeated from certain clients that ring the police a lot and there’s drug and alcohol issues or whatever—I find the response a bit blasé sometimes from those organisations, or a woman desperate and goes to the emergency department to present with a child and they just turn her away. And then the shelters are full, the housing is limited so I find that really hard. We’re Monday to Friday nine to five so that limits what we can do. We’ve only got the resources we’ve got, we’re not a crisis service so then when we go to use or direct our clients to that crisis service, and they’re turned away they stop going there. They go, ‘What’s the point?’”*

(ACCHO Management/Team leader)

ACCHO staff described accountability to community and connected to this there was a sense that ACCHOs were responsible for clients who have a negative experience at another service.

*“Because you refer someone, or you drop someone off to a service and they get a shit service, or they don’t even get past the receptionist. Of course, it comes backs on you because we do have a responsibility to improve access, you know. I mean we can’t do everything, but if they’re not getting a decent service, we need to do something about that”*

(ACCHO Management/Team leader)

#### 3.1.2. Making Partnerships Work

While there was consensus that partnerships were essential for achieving good outcomes for clients, they took considerable resources (time, financial and staff) to establish, maintain and navigate. Reaching across formal and informal networks was essential, and this could be challenging for new staff who did not have established links to community and other organisations. One of the challenges around maintaining partnerships was keeping up to date with the turnover of staff in partner organisations, as well as changes to funding and introduction of new programs.

*“I think we could do more in terms of the NGOs, I think a lot of us rely on who we know in services and then people move around. I tend to follow the clinician more than the service because I know who’s going to respond and who’s going to do what so it’s just keeping up to date with that… we just don’t have time to get out or even know where they’re doing what they’re doing. Because stuff changes quickly, funding changes and it’s hard to keep up.”*

(ACCHO Management/Team leader)

*“So, yes, it can be quite complicated, the relationships—and it can be quite time consuming as well because it’s just a constant thing that you’ve got to give attention to. You can’t have an MOU [memorandum of understanding] and put it on the shelf and think ‘oh, well, sweet we’ve got that under control’—because people change, things change and you’ve actually got to have these organic partnerships, that take a huge amount of work, huge amount of work”*

(ACCHO Management/Team leader)

Much of the time and resources that go into developing partnerships are not accounted for in KPIs set by funding bodies; in particular, the significant resources and benefits that come from good communication, transparency and developing trusting relationships over time.

*“Communication is—once again, it’s not rocket science, is it, but we’ve had a really great outcome recently, with incredible communication with Justice Health….They’re just really good outcomes, like incredible communication over what services, and the women are just going, “This is awesome,” because she’s been listened to, she knows what’s happening there’s transparency—transparency’s a big one—letting women know what you’re doing …. And that’s probably benefits of a multidisciplinary team being together and making sure everyone knows what’s happening, that can be really good”*

(ACCHO Team member)

*“And that’s based on the historical context, and we have a very good relationship with Corrective Services here, and the magistrate understands all that, so if there’s a (ACCHO) worker down there at the courthouse, he will look more favourably on (ACCHO) support with this particular family, because children’s court they’re closed sometimes, but he has allowed (ACCHO) people in there to support the family; he’s understanding. Not many courts will do that, but our local court does.”*

(ACCHO Management/Team leader)

#### 3.1.3. Acts of Resistance 

ACCHO staff described acts of resistance in partnerships, which were frequently in response to unjust processes or treatment that were counter to decolonisation. At times, this meant having frank conversations and “*pushing back*” when ACCHOs felt that community was not at the centre of the partnership and that clients’ needs were not being met. Often this process involved ACCHO staff challenging power differentials and confronting racism toward clients and staff, which represents resistance and is a key component to decolonisation and “*pushing back*” against structures of Whiteness and systemic racism.

*“So, the (NGO), about two years ago they setup a 1300 number for people to ring for food assistance and financial assistance. You can be on hold there for over an hour and then all of a sudden, they’ll say, ‘I’m sorry, we’re unable to assist you today, please ring back tomorrow.’ So that infuriated me, let alone someone that’s starving, poor mental health, whatever. So, I rang and complained to head office and said, ‘This just isn’t acceptable. I haven’t been able to access your service for eight months’ and I said, ‘And I’m leaning on other services and I don’t think that’s fair.’ So, I then got the direct number for the person down here. Now I just ring them—you really have to be a proactive worker to want to go out on a limb and do things like that”*

(ACCHO Team member)

Pushing back was also seen as a mechanism for strengthening partnerships, which ultimately achieves better outcomes for clients and community and disrupts power differentials as part of decolonisation.

*“There’s some pretty solid work that we’ve done for, probably, for a number of years with certain government departments…. where that’s made a huge impact for community. That’s probably come from pretty bad places to very positive relationships now. So, that’s really important because, again, it’s that first response stuff. But we do have, you know, fractious relationships, too, with some services we believe are not delivering good services. And I suppose we have never been backward in coming forward in saying what we think about that. But always with, you know, through decolonisation lens… and I’m not saying that you need to be arguing with everyone all the time either. Because that often, can be counterproductive but yeah, I think we’re pretty on par in knowing that partnerships are important because we know, if we can influence and have impact on other service providers then that—what’s going to happen is better outcomes for the women”*

(ACCHO Management/Team leader)

Often, the issues around pushing back against the poor treatment of clients in mainstream services intersected with how ACCHO staff perceive these services respond to and understand trauma. ACCHO staff described mainstream services as having reductionist views of trauma that are not grounded in meaningful understanding of transgenerational and cumulative trauma. In practice, these views serve to problematise and pathologise individuals’ expressions of trauma and reactions to oppressive systems, such that clients were viewed as difficult or non-compliant. In these partnerships, staff considered their role had dual purposes, first to advocate for clients and second to strengthen cultural awareness in mainstream services and staff.

*“So, she’s trying to get her finger in all of those pies, to make sure that they’re engaging our women in a culturally appropriate way rather than alienating them. You know, you can’t speak that sort of language to some of our women. Because I wouldn’t understand it. You know, the language and the terminology that they use when they’re referring to—for the impacts of trauma or whatever, you need to say it as it is”*

(ACCHO Management/Team leader)

While cultural competency training is standard practice in ACCHOs and mainstream organisations, ACCHO staff reported that in practice, cultural competency was significantly lacking among many mainstream service staff. As a result, ACCHO staff felt tasked with fostering cultural competency among partnering services and their staff, and this could be a burden at times in the setting of multiple responsibilities.

*“That’s a lot what I do with the government organisations, is to talk about the cultural stuff that’s going on and the intergenerational trauma and things like that. So, a lot of service providers ask me questions because they feel comfortable enough to sort of ask me those types of questions in private”*

(ACCHO Team member)

Given the value placed on interagency partnerships achieving good outcomes for clients and community, there was consensus that more needed to be done to strengthen these partnerships. However, it was evident that a new approach which shifted the responsibility of ensuring community wellbeing, safety and justice from ACCHOs to a shared responsibility for all sectors to jointly enact a ‘decolonisation’ agenda was needed.

### 3.2. A New Approach to Decolonising the Workforce

There was strong consensus that workforce capability is key to working effectively with and for Aboriginal and Torres Strait Islander peoples and communities. Accordingly, non-Aboriginal colleagues and interagency partners who were culturally competent and worked in a culturally safe way were highly valued by Aboriginal and Torres Strait Islander staff and were viewed as allies: “They walk beside us”. Moving beyond cultural competency, the data that explored issues around decolonisation and workforce capability amalgamated into three sub-key themes: (1) a skilled and capable workforce across sectors; (2) decolonisation: more than just ‘cultural competency’; (3) understanding and reflecting on white privilege.

#### 3.2.1. A Skilled and Capable Workforce Across Sectors

Decolonising approaches should be an important component of workplace competency; however, most workplaces provide only cultural competency training. To address this, Waminda has developed programs for non-Aboriginal staff (approximately 25% of all staff) that go beyond cultural competency training and engages them in an individual and collective process of decolonisation. The programs combine cultural mentoring and participation in an ongoing group ‘allyship’ program: Imperfect Allies, which focuses on understanding colonisation, white privilege, and taking responsibility for working through a decolonisation lens.

*“So, it is a process that’s actually really helping, and what it is, it’s taking responsibility. So, the white people have to take responsibility for white people here. And you talk to a lot of the Aboriginal senior staff here, and they’re just relieved, that they don’t have to sort us out. Like, it’s our responsibility to confront that”*

(ACCHO Management/Team leader)

The positive impacts of this workforce decolonisation intervention at Waminda led the staff to consider how this model could be adapted to work with their external partners to generate systemic change. Waminda, initially worked with the local health district to deliver decolonisation workshops.

*“And so, having these sessions with staff, with the Health District, where we looked at, walking in two worlds and looking at traditional way[s] of people living but reflecting on, well, what’s changed, what has colonisation actually done. So, it’s not all that warm and fuzzy stuff and this is a didg [didgeridoo]… it’s really quite different. But a big part of what happens is that looking at colonisation, transgenerational trauma, very much about white privilege and white fragility and really breaking that down. So that’s had a massive impact”*

(ACCHO Management/Team leader)

While this worked well with health partners, it did not address many of the issues that persisted in partnerships with other external government and non-government organisations across justice, education, social services and particularly partnerships around children in out of home care.

*“Until you look at decolonisation and systemic racism and where this has all come from and your white privilege, you can’t do anything else…. I said: ‘If people aren’t up for this discussion, the problem is bigger than you think.’ And I said, ‘We won’t be partnering in community with anybody who doesn’t see this as a major issue or the issue’”*

(ACCHO Management/Team leader)

Waminda sought to address this within a strategic leadership group that had been established in the Illawarra Shoalhaven region, with members from the Department of Communities and Justice, Health, Education and NGOs to work collaboratively to improve outcomes for Aboriginal and Torres Strait Islander families. Within the strategic group, it was agreed that Waminda would deliver a one-day decolonisation workshop to members of the Illawarra Shoalhaven Department of Communities and Justice Strategic Leaders Forum.

*“You know, people didn’t know what decolonisation was at all and that wasn’t even a judgement by us, it’s just, like, wow, that’s not even talked about”*

(ACCHO Management/Team leader)

*“We’ve had to think about what we do about that, in a bigger picture. So not just that worker to worker, people experiencing that prejudice or that racist approach, but really trying to look more systemically, I suppose. And so out of that very much, looking at that systemic racism and institutional stuff, we have been really working a lot at that level and having a lot of conversations with high levels of government”*

(ACCHO Management/Team leader)

#### 3.2.2. Decolonisation: More Than Just ‘Cultural Competency’ 

The workshop approached colonisation in terms of the historical as well as the local context, including the ongoing impacts of transgenerational trauma, systemic retraumatisation, structural and institutional racism and injustices. Participants were very receptive to the messages delivered, yet also recognised the challenge inherent in enacting real systemic change.

*“The take home messages are that colonisation is alive and well and everything that we’ve designed in our system is not designed with Aboriginal people in mind, and that is so massive, how do you make a change that’s going to be able to meet the objectives … and be effective and respectful for people on the ground”*

(Decolonisation workshop participant)

*“Oh my God, every single person in Australia needs to attend that training, but they’re not going to, so then how do we do this?”*

(Decolonisation workshop participant)

One of the key components of this workshop was the personal stories shared by Waminda staff and Dharawal Elders, which participants found highly engaging. While participants described the content as “confronting” and “challenging”, they also felt that this is what elevated the workshop from previous training and felt that the stories shared were “very powerful”. Stories were important for expanding participants’ world views by demonstrating with real world examples how organisations and systems privilege Whiteness and how this impacts the local community.

*“So, I think that it’s the life stories— so having somebody standing up in the room …. sharing their stories is a really, really powerful tool”*

(Decolonisation workshop participant)

*“I think it (the workshop) focused less on Aboriginal cultural practice. I think most cultural awareness training that I’ve been to has a really strong focus and is often very locality based—and it’s often really focused on sharing the local culture and the local history. So, I guess, for me, this one moved away from that. It talked a lot about Aboriginal history, in a much broader context as colonisation, which is something that I studied at uni and had the awareness of, but I guess this workshop really personalised it a bit more for people and the context or the impact on particularly some of the workers (Wamimda staff) who spoke”*

(Decolonisation workshop participant)

*“I particularly enjoyed the sisters (elders) that came from the Illawarra, and they were able to give us some really deep, local knowledge and context. So, in terms of a level of investment in improving our knowledge, understanding and engagement, I thought it was very, very high [quality]”*

(Decolonisation workshop participant)

#### 3.2.3. Understanding and Reflecting on White Privilege

The content that focused on white privilege was very well received by participants. For some it was new knowledge and terminology. Participants affirmed that white privilege is not well understood or accepted in Australia and they had reflected on this considerably since the workshop. Some participants had shared this new knowledge with their co-workers, friends and family.

*“I’m really getting a lot out, at the moment, around the whole focus on white privilege. I think there needs to be more of that education, and that’s what I’ve been educating my white friends about. Because they don’t know—or let’s just say—they don’t think there’s any such thing as white privilege”*

(Decolonisation workshop participant)

Participants consistently felt that this component was a key strength of the workshop and there was agreement that they would be aiming to take this knowledge back to their organisations to inform their work with young people and families. In this way, leaders recognised their own position as change makers in their organisation and that it was their responsibility to bring a decolonisation lens to their organisation and to ensure that the workforce are skilled and capable of working in this way.

*“I think because I manage staff, I’ll have it always in the back of my mind that this is a focus area for us, that I need to have my staff’s understanding of those concepts in regard to white fragility as well as white privilege in the back of their mind in regard to whatever they’re doing with their target group of young people”*

(Decolonisation workshop participant)

### 3.3. Decolonising Partnerships

Unlike previous cultural competency training that participants had attended in their organisations, there was a sense that the workshop was more than a one-day training event. Instead it represented the beginning of a new way of thinking about decolonisation and a commitment among regional leaders to working collaboratively to enact systemic change. Two key sub-themes emerged: (1) shared experience and common language; (2) responsibility and change.

#### 3.3.1. Shared Experience and Common Language 

The workshop participants comprised of leaders in their organisation and across the region; however, it was widely acknowledged how valuable it was for them to spend time collectively listening and reflecting.

*“When you think about that group, incredible group of leaders and many leaders for a really long time, we’re all much more used to talking than listening … having the opportunity to have our opinion …but I think this was a real opportunity to spend some time listening and reflecting”*

(Decolonisation workshop participant)

*“So it was very good. I mean, it took me a while to actually get into that headspace. All of us in that room are really, really busy people and there’s so many demands. But once I managed to get over that I really got a lot out of it”*

(Decolonisation workshop participant)

Bringing the strategic leadership group together for the workshop generated a shared experience and common language, which many participants felt would be integral to building collaboration and enabling identification of strategic priorities and actions going forward.

*“You know, a lot of people sitting in that room, I don’t see much at all, but just having that shared knowledge and knowing, okay, this is not just our organisation that’s thinking through these issues, there are others, and we either need to connect more or coordinate effort more strongly …. and just getting that sense that we’ve now got a bit of a shared language in one sense. I know that’s a big phrase because the issue is not a new one, but the fact that we’ve had that shared experience”*

(Decolonisation workshop participant)

The shared experiences and learning from the workshop were key to generating this collective responsibility to drive systemic change regionally and ensuring that Aboriginal and Torres Strait Islander voices are centred through a decolonising approach.

*“I’m sure that everybody in that room had a strong commitment to positive outcomes for Aboriginal communities and Aboriginal families. There’s no doubt. I’m absolutely certain that many, many people in that room already worked well with Aboriginal communities, but I just feel like it sort of draws a line in the sand that we all can agree on, and so commonality around language and objective”*

(Decolonisation workshop participant)

*“Having that workshop was something that we could all agree on, was necessary to build a more collaborative focus for that leadership group ….So this, I think, will enable, hopefully, a strategic focus on how the region or the district applies itself and how we can make sure that our programs in that district are doing that work to the best that we can and how we are shaping our services”*

(Decolonisation workshop participant)

#### 3.3.2. Responsibility and Change 

At an individual level, participants reported that everyone has *“a role to play”* in addressing structural and institutional racism, and that this begins with *“calling out”* racism and seeking to educate friends, family and colleagues about the multiple ways in which racism and white privilege are enacted and normalised in Australia.

*“It’s all of our responsibilities to think about what we can all contribute, and it’s a community issue, that we all need to have a level of ownership of …as well as a personal one—but I thought it was clever how it [the workshop] enforced or emphasised the collective responsibility we all have in our respective positions, to pay attention, to deal with this issue”*

(Decolonisation workshop participant)

Participants described multiple impacts from the workshop at the organisational level. One of the most critical was a commitment to ensuring that all staff, especially non-Aboriginal staff, are responsive and skilled in their work with families. Key to this was introducing some of the concepts from the workshop as a focus in the organisation, including a meaningful understanding of systemic drivers of trauma and racism.

*“But recognising that the trauma is with the person and with the family and with the community and stays there and that when you’re working with them you need to work in a way that recognises that”*

(Decolonisation workshop participant)

*“So I’ve kind of really pulled back from the whole launch idea now as well, and just having a think about how we might do that in the teams and have deeper conversations more about structural racism and some of the concepts that we were really getting exposed to in the workshop. And, doing more of that to make the plan more personal for each person, rather than it just being one big event that’s engaging at a fairly superficial level”*

(Decolonisation workshop participant)

There was also acknowledgement that the responsibility to understand and address these issues at an organisational level must not translate to relying too heavily on Aboriginal and Torres Strait Islander staff for cultural competency. Instead, participants recognised their role as leaders to ensure that all staff take responsibility for working responsively and effectively with families and for ensuring that the Aboriginal and Torres Strait Islander workforce are well supported and not over-burdened.

*“One of the things I’ve done since the workshop, is we have an Aboriginal staff group that meet with the managers and that’s around …how we strengthen our work with Aboriginal families and also support our Aboriginal staff as well, and I’ve just really noticed our managers in that group are really relying on those Aboriginal people in that group to lead it, as opposed to the other way around”*

(Decolonisation workshop participant)

*“Some of the key things for us to think about is being careful not to overly rely on our Aboriginal staff. Yeah, around our education and really taking responsibility as—we have Aboriginal leaders but as non-Aboriginal leaders within our organisation as well”*

(Decolonisation workshop participant)

Overwhelmingly participants from the regional leaders forum reported that the workshop affirmed some of what they already knew about the history of colonisation, oppression, injustice and transgenerational trauma but provided new understanding around what it means to take collective responsibility for having families at the centre of their work.

*“What I think it will do is continue to build momentum around Aboriginal families being a priority for our district... So, I think it will cement that and because it was so powerful that will stay with people and will give them the incentive to keep planning around this”*

(Decolonisation workshop participant)

*“I think the main benefit of the workshop is that it gets everybody on the same page. So, I think from (Organisation) perspective their work is going to focus more on working with Aboriginal communities and Aboriginal families. And I think as a strategic group leading that direction, I think it’s really important that we all view that from the same standpoint”*

(Decolonisation workshop participant)

## 4. Discussion

**Figure 2:**
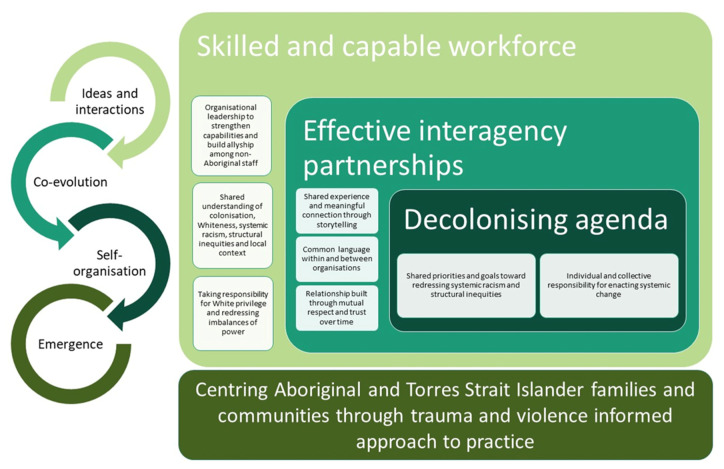
ACCHO-led model of systemic decolonisation that centers families and communities through: (1) enhancing the skills and capabilities of the workforce and (2) effective partnerships through meaningful connection, common language and relationships built through trust and respect over time to generate a shared decolonisation agenda. The circular arrows depict this process from a complexity perspective.

This case study describes firstly the context in which ACCHOs build interagency partnerships, which requires navigating complex funding, discordant priorities and power differentials. Secondly, this case study demonstrates an adaptive response to these partnership challenges, in which Waminda has harnessed the power of activism to reorient interagency partnerships toward working collaboratively through a decolonisation lens. From a complexity perspective, Waminda shared knowledge and generated interaction that initiated the co-evolution of this deeper understanding of decolonisation, which was a critical driver in self-organisation of interagency partnerships at a regional level to share responsibility for generating emergent systemic change toward disabling power structures that perpetuate inequity (Figure 2). This approach offers several important learnings and implications for how a shared experience and common language among interagency partners catalyses individual and collective responsibility to pursue a decolonisation agenda.

### 4.1. Co-Evolution: Language Shapes Responses

In a socially connected system, relationships, behaviours and processes *co-evolve* as people within the system interact, share ideas and knowledge, and within this, certain people or collectives will be highly influential [22]. In this model (Figure 2), “interactions” include networks, cliques, hierarchies and heterarchies [24] while “ideas” encompass beliefs, conceptual understandings, thoughts and plans. Viewed through the lens of complexity, the present results elucidate how ideas that are held and shared by the workforce are critical in generating local rules, processes and collective behaviours resulting in positive or negative outcomes and unintended consequences [23]. However, the workforce is highly diverse in terms of knowledge, motivations, history and lived experience of individuals, and as such ideas and interactions within organisations and beyond are highly variable. 

Indeed, the present results indicate that these interactions can be fraught in the face of discordant world views, particularly around institutional racism, white privilege and transgenerational trauma, which can have unintended consequences for ACCHOs, their staff and communities. Therefore, it is imperative that non-Aboriginal staff have the capacity and language to work competently, respectfully and effectively with Aboriginal and Torres Strait Islander organisations, staff and families. To address this, cultural competency and cultural awareness training has become commonplace and indeed mandatory in most organisations. 

Despite the widespread uptake of cultural awareness and competency training in mainstream organisations, substantial shortcomings of such approaches have been identified including lack of accountability and reluctance to challenge structural inequalities [34,35,36] Furthermore, cultural competency frameworks in Australia have been criticised for their lack of attention to trauma and racism experienced by Aboriginal and Torres Strait Islander people [37]. The decolonisation approach developed by Waminda addresses these shortcomings by expanding the scope of what it means to be culturally capable to include knowledge and actions around structures of Whiteness and privilege, as well as how this has impacted historically through colonisation and how it continues to impact through institutional racism, structural violence and trauma. 

Central to Waminda’s approach, was sharing lived experience knowledge to demonstrate and shape understanding of how colonisation impacts and has impacted local communities and families. The use of storytelling allows people to share knowledge aligned with their cultural integrity, which is critical to decolonisation by centring the voices that racial oppression seeks to silence [38]. Storytelling within Waminda’s decolonisation workshop generated counter narratives that were important in exposing the ways in which casual, overt and institutional racism are experienced in contemporary Australian society. This demonstrates the power of storytelling as a mechanism of decolonisation, which in the present study, underpinned the *co-evolution* of shared understanding among Waminda and interagency partners, particularly around colonisation, racism and Whiteness.

### 4.2. Self-Organisation: New Ways of Working 

Before the decolonisation workshop, there was a lack of common language around Whiteness between Waminda and interagency partners. There was also discordance in how ACCHOs and other organisations understood (or acknowledged) white privilege, which had an impact on their capacity to work together effectively with and for Aboriginal and Torres Strait Islander families. The workshop led by Waminda was a catalyst to changing this and shifting the focus of regional collaboration toward *self-organising* around a shared decolonisation agenda. A key driver of this was the interdependencies between how people understand and speak about Whiteness and how this shapes their actions and responses. In white hegemonic nations such as Australia, the acceptance of Whiteness as the cultural norm maintains the balance of power in favour of white people and institutions [15,39]. Related to this are critiques of the concept of white privilege, which as Cabrera [40] (p. 78) asserts, may do a disservice to anti-racist actions because “instead of engaging issues of racism, white people frequently search in their personal histories for narratives of struggle and then use them to downplay the significance of white privilege”. Cabrera [40] suggests that “White immunity” is a more nuanced and effective term for conveying the invisible cloak of advantage that protects white people from systems and structures of oppression and racialisation. 

This invisible positioning of Whiteness means that white people benefit in seen and unseen ways from hegemonic Whiteness. The lives of white people are unburdened by issues of race and the largely unacknowledged privilege of Whiteness. Countering hegemonic Whiteness is central to addressing all forms of racism. Despite this, cultural competency training in the workplace or educational setting very often does not directly address racism much less hegemonic Whiteness or white privilege [36]. Indeed, confronting white privilege and Whiteness can trigger reactions and defence, which is frequently termed “white fragility” [36]. White fragility can take many forms, and DiAngelo [36] describes it as emotional intolerance for confronting issues of racial stress, which can manifest emotionally (guilt, fear, sadness, anger) and behaviourally (silence, arguments, avoidance). Such responses centre white peoples’ experiences and serve to maintain white supremacy. However, criticisms of white fragility, relate to the underlying assertions of unconscious bias, which do not sufficiently hold white people to account [41]. Indeed, it has been argued that this approach needs to be decolonised, and accountability and action must be embedded to bring about anti-racism transformational change [41]. This was a key strength of Waminda’s approach, which was embedded within broader collective actions, and importantly, reflecting on white privilege and fragility were undertaken in the workshop through the lens of decolonisation rather than unconscious bias.

In the present study, participants’ reflection on their own white privilege was critical in exposing the multiple ways in which Whiteness emerges from social processes and practices that centre white people, their beliefs, experiences, voices and rights. The impacts of having language and understanding around white privilege were evident in multiple levels of *self-organisation* toward anti-racist actions including “calling out” racism and “taking responsibility”—and importantly around new organisational processes and directives. A key example was management indicating their role as leaders who take responsibility for ensuring this competency within their own staff and “not overburdening Aboriginal and Torres Strait Islander staff”. This over-reliance on Aboriginal and Torres Strait Islander staff to lead cultural competency in organisations is not a new issue [37]; yet here we see how it intersects with white privilege and how reflecting on one’s own privilege is a catalyst for taking responsibility rather than delegating responsibility for change.

### 4.3. Emergence: The Butterfly Effect

While complex social systems are constantly evolving and adapting, the emergent properties and changes are rarely quantifiable or observable. However, it is important to note that seemingly small changes in interactions and behaviour can give rise to more profound adaptations in the system as a whole [22]. In this way, there is a “butterfly effect”, whereby co-evolution of actors within the system, leads to subtle changes in interactions that ‘reverberate’ and self-organise into new behaviours, which over time can lead to profound changes within the system as a whole [22]. This was evident in the present results, where discordant interagency interactions compelled Waminda to develop and drive a decolonisation agenda with interagency partners in order to secure better outcomes for families and communities. The decolonisation workshop was a vehicle for realising this change and sowing the seeds for establishing shared priorities and goals for working in partnership to redress systemic racism and structural inequities. 

Decolonisation is a personal journey that is ongoing and implores people to centre the world view of the ‘colonised’ and to explore how their own world view, experiences and actions are shaped by colonisation, and to consider who benefits, and how, from colonial processes and injustices [6,7,42,43]. The workshop was a catalyst in decolonising the self, which is a critical task requiring us to recognise “how we have all been affected by colonisation” [5] (p. 291). Positive interactions and feedback were conduits to delivering new conceptual knowledge around Whiteness, privilege and racism. Inherent within this decolonisation process is the interdependencies between language and beliefs and how this shapes our responses. Recognising these interdependencies saw Waminda shift from “pushing back” individually with partners, to working collaboratively to enhance the knowledge and capabilities of their workforce, and ultimately to reorienting partners toward enacting systemic change that centres Aboriginal and Torres Strait Islander families through decolonisation actions.

### 4.4. Implications for Practice, Policy and Future Research

At the individual level, there is recognition that leaders are change makers in complex systems, but this research also emphasises that all individuals within the system have power to initiate change that can have far reaching systemic impacts. While it is undeniably everyone’s responsibility to enact decolonisation of the self, health organisations and social systems must support their workforce to embark upon this journey by enabling organisational policies and partnerships with ACCHOs. We strongly emphasise that it is not appropriate for mainstream services and/or non-Aboriginal people to attempt this in isolation from Aboriginal and Torres Strait Islander communities—working in partnership and centring the experiences of communities is key to decolonisation. Waminda’s approach to decolonisation has demonstrated that ACCHO leadership is integral to this process and there is power in sharing lived experience and counter-narratives. Further, within decolonisation processes or interventions, Aboriginal and Torres Strait Islander people and organisations must be appropriately acknowledged and compensated for their leadership, partnership and knowledge.

At the organisational level, decolonisation in health and social services can be strengthened through the integration of *trauma and violence informed care* [17]. While there is widespread uptake of trauma-informed approaches to addressing complex trauma and traumatic stress, trauma and violence informed care is a broader approach to addressing the traumatic impacts stemming from interactions with colonial systems, including structural violence and racism. Thus, trauma and violence informed care is a mechanism to counter the tools of colonisation that structurally and individually have enabled ongoing oppression and dispossession of Aboriginal and Torres Strait Islander peoples. Future research should be directed toward understanding how this approach could be integrated effectively to deliver high quality care with regard for the cultural safety of people attending mainstream health and social services. This would best be achieved through Indigenous research methods and centring the voices and experiences of Aboriginal and Torres Strait Islander communities and peoples. Additionally, future research could investigate the dynamics of collective experiences to better understand how these are powerful and can be harnessed to impact people, organisations and systems.

At the systems level, decolonisation needs to be embedded within policy. The landmark *Bringing Them Home* report called for a “trauma-informed public policy environment” [44] (p. 30). We endorse this, and also call attention to policy that references racism, colonisation, and trauma yet does not adequately embed these concepts within policy directives or in actions to implement change [45]. We assert that addressing racism and trauma within complex health and social systems will be constrained unless decolonisation approaches are embedded within Aboriginal and Torres Strait Islander led enabling policy directives. An implementation science approach is needed to support the uptake of decolonisation within policy and the translation of decolonisation knowledge into practice. 

### 4.5. Strengths and Limitations 

The case study method is appropriate as it enables identification of meaningful experiences, practices and relationships, told through a narrative arc. By combining this with complexity thinking as a framework for interpreting findings, we can explore the richness in how people (individually and collectively) creatively adapt and develop locally tailored solutions to generate systemic change [20,21,46]. A strength of our approach has been the triangulation of multiple data sources across multiple time points and modes of data collection. This enabled us to gather a rich and layered understanding of how the issues intersect locally and politically from diverse standpoints. 

A primary limitation relates to who participated—while we have sought the perspectives of Non-Aboriginal and Aboriginal and Torres Strait Islander people, it is possible that self-selection bias has meant that those who participated were more inclined towards an anti-racist stance. We also do not have feedback from all participants who attended the decolonisation workshop. It is possible that those who gave feedback viewed the workshop more positively than those who did not respond. Also, our follow-up period was two months post workshop. It is not clear from our findings how the workshop has impacted the long-term goals and actions of the organisations. This was beyond the scope of the present research; however, we suggest that a robust evaluation of Waminda’s decolonisation approach would indicate the applicability and scalability of this intervention for similar contexts.

We have focused on the experiences of communities along the Central and South coast of NSW offering substantial insight into the experiences of the NSW ACCHO workforce and how they embed decolonisation in partnerships to drive systemic change. Given the deliberate sampling, the interpretation of the results is limited to this case study. While this cannot be generalised too broadly, our findings have relevance in Australia and globally in other settler colonial contexts in which decolonisation could strengthen the health and social sectors.

## 5. Conclusions

Health and social services are complex systems and implementing changes in policy and practice are equally complex and multi-faceted. These complex systems operate within the context of colonisation, which in itself is a complex systems dynamic. Achieving systemic change requires effective interagency partnerships, however these partnerships can be fraught with issues of power imbalance, competing priorities and discordant worldviews, all of which are manifestations of a complex social system that sits in a colonised context. To counter this, Waminda has driven a process of collective learning and reflecting with partners to generate a common language around colonisation and structures of power, racism and Whiteness. The decolonisation workshop was a catalyst for ideas and interactions, which *co-evolved* into a shared understanding and was a critical driver of *self-organisation* or new ways of working within organisations and in partnerships. This was the basis for an *emergent* collective decolonising agenda toward redressing colonising structures of power and centring families. Beyond enhancing workforce capability and strengthening partnerships in terms of knowledge, language and understanding, Waminda’s approach represents a call to action for the Australian health and social services sector, and in particular for organisational leaders to take responsibility for enacting change and decolonising themselves and their organisations.

## Supplementary Materials

The following are available online at https://www.mdpi.com/1660-4601/17/20/7363/s1.
