# Supplementary material for: Trauma and Violence Informed Care Through Decolonising Interagency Partnerships: A Complexity Case Study of Waminda’s Model of Systemic Decolonisation
Source: Int J Environ Res Public Health. 2020 Oct 9;17(20):7363. doi: 10.3390/ijerph17207363 (PMC7601198; doi:10.3390/ijerph17207363)
Supplement: Supplementary file 1 [file ijerph-17-07363-s001.pdf]

Table 1. First Response Decolonisation Workshop Interview Guide.

1.	What is the main role of your organisation?
2.	What is your role within the organisation?
3.	What services relating to Aboriginal women/families do you provide?
4.	Do you/your organisation work with ACCHOs? If yes, please describe how you/your
5	organisation works with (ACCHO).
6	How did you find the Decolonisation workshop?
7	What worked well/did not work well (in the workshop)?
8	What do you think could be improved/changed?
9	Do you think this will inspire any changes in your work/organisation/team? If so, please describe. If not, why?
10	Has there been any other impacts? Please describe.
11	Was it different from other types of cultural awareness training? Could you please describe how it was different/similar?
12	Do you think this will influence the collective work of the regional strategic group? If so, please describe. If not, why?
13	What is your understanding/views on trauma informed care/culturally safe care?
14	What types of training have you had in trauma-informed care/culturally safe care?
15	Are there any other comments that you'd like to make?
